# The Predictive Value of Low Muscle Mass as Measured on CT Scans for Postoperative Complications and Mortality in Gastric Cancer Patients: A Systematic Review and Meta-Analysis

**DOI:** 10.3390/jcm9010199

**Published:** 2020-01-11

**Authors:** Alicia S. Borggreve, Robin B. den Boer, Gijs I. van Boxel, Pim A. de Jong, Wouter B. Veldhuis, Elles Steenhagen, Richard van Hillegersberg, Jelle P. Ruurda

**Affiliations:** 1 Department of Surgery, University Medical Center Utrecht, Utrecht University, Heidelberglaan 100, 3584 CX Utrecht, The Netherlands; a.s.borggreve-2@umcutrecht.nl (A.S.B.); r.b.denboer@students.uu.nl (R.B.d.B.); gijs.vanboxel@googlemail.com (G.I.v.B.); r.vanhillegersberg@umcutrecht.nl (R.v.H.); 2 Department of Radiation Oncology, University Medical Center Utrecht, Utrecht University, Heidelberglaan 100, 3584 CX Utrecht, The Netherlands; 3 Department of Radiology, University Medical Center Utrecht, Utrecht University, Heidelberglaan 100, 3584 CX Utrecht, The Netherlands; p.dejong-8@umcutrecht.nl (P.A.d.J.); w.veldhuis@umcutrecht.nl (W.B.V.); 4 Department of Dietetics, University Medical Center Utrecht, Utrecht University, Heidelberglaan 100, 3584 CX Utrecht, The Netherlands; e.steenhagen@umcutrecht.nl

**Keywords:** gastric cancer, muscle mass, prognostication, sarcopenia, gastrectomy, CT scan, risk factors

## Abstract

Risk assessment is relevant to predict outcomes in patients with gastric cancer. This systematic review aimed to investigate the predictive value of low muscle mass for postoperative complications in gastric cancer patients. A systematic literature search was performed to identify all articles reporting on muscle mass as measured on computed tomography (CT) scans in patients with gastric cancer. After full text screening, 15 articles reporting on 4887 patients were included. Meta-analysis demonstrated that patients with low muscle mass had significantly higher odds of postoperative complications (odds ratio (OR): 2.09, 95% confidence interval (CI): 1.55–2.83) and severe postoperative complications (Clavien–Dindo grade ≥III, OR: 1.73, 95% CI: 1.14–2.63). Moreover, patients with low muscle mass had a significantly higher overall mortality (hazard ratio (HR): 1.81, 95% CI: 1.52–2.14) and disease-specific mortality (HR: 1.58, 95% CI: 1.36–1.84). In conclusion, assessment of muscle mass on CT scans is a potential relevant clinical tool for risk prediction in gastric cancer patients. Considering the heterogeneity in definitions applied for low muscle mass on CT scans in the included studies, a universal cutoff value of CT-based low muscle mass is required for more reliable conclusions.

## 1. Introduction

Gastric cancer is the third most common cause of cancer-related deaths in the world. Despite recent improvements in treatment, the outcomes of patients diagnosed with gastric cancer remain poor [1,2]. The main curative therapy for advanced gastric cancer without distant metastases is surgical resection with perioperative chemotherapy or chemoradiation [2,3]. Unfortunately, this procedure is accompanied by severe postoperative complications such as pulmonary complications (13%), cardiac complications (6%), intra-abdominal abscess (4%), and anastomotic leakage (3%), resulting in a considerable postoperative mortality percentage of 5% [4]. To date, tumor-specific factors such as histological subtype, depth of tumor invasion, size, and metastases (lymph nodes as well as distant metastasis) have been identified as important risk factors for a poor prognosis in patients with gastric cancer, as well as patient-specific factors such as low body mass index (BMI) [5,6,7,8,9]. Accurate prediction of a patient’s prognosis may impact clinical decision making and patient management, but remains unsatisfactory to date.

Sarcopenia is a clinical condition defined as the generalized loss of skeletal muscle mass and strength [10]. It mostly occurs with advancing age, but can also be induced by inactivity, malnutrition, and diseases such as cancer [11]. The effect of low muscle mass on postoperative complications and mortality in gastric cancer has been studied in numerous studies, but yielded equivocal results [12,13]. Several methods have been proposed for the assessment of muscle mass, with measurement of the cross-sectional muscle area at the level of the third lumbar vertebra (L3) on computed tomography (CT) scans being the most commonly used [14,15]. Within the CT-based method, several cutoff values for muscle area are applied to define low muscle mass. Hence, the clinical impact of low muscle mass for gastric cancer patients remains undefined. This systematic review and meta-analyses aimed at summarizing the predictive value of low muscle mass as measured on CT scans for postoperative complications and mortality in patients with gastric cancer. 

## 2. Methods

This systematic review and meta-analysis followed a predefined protocol registered with PROSPERO (CRD42018091573) [16] and adhered to the Preferred Reporting Items for Systematic Reviews and Meta-Analyses (PRISMA) guidelines [17].

### 2.1. Search Strategy

A systematic PubMed and Embase literature search was updated up to 24 August, 2019, to identify all studies reporting on muscle mass as measured on CT scans in patients with any form of gastric cancer. Search terms that were used to identify such articles were “gastric cancer”, “muscle mass”, “computed tomography”, and synonyms (see supplementary material for the exact search strategy).

All articles reporting on muscle mass as assessed on CT scans in gastric cancer patients and published in English, regardless of study design and applied definition of low muscle mass or duration of follow-up, were included and reviewed. The reference lists of all included studies were hand-searched in order to identify other potentially relevant studies. Congress abstracts, case reports, and reviews were excluded. To create a homogenous group of inclusions regarding tumor stage, studies reporting on in situ tumors and palliative metastatic disease were excluded. Because (severe) postoperative complications and (disease-specific) mortality were the most common and most clinically relevant outcomes in the search, we excluded studies with other outcomes. Subsequently, duplicates were removed and article selection based on title and abstract was performed by two researchers (R.B.d.B., A.S.B.). Any disagreements were resolved by discussion between the two researchers (R.B.d.B., A.S.B.). 

One researcher (R.B.d.B.) extracted data, including author, year of publication, continent of origin, study design, type of gastrectomy, surgical approach, definition and normalization method of muscle mass, number of patients, prevalence of patients with low muscle mass, characteristics of the patient population (age, sex, tumor stage, and duration of follow-up), and the reported outcomes. A second researcher verified all extracted data (A.S.B.). 

### 2.2. Study Outcomes

Outcome measures were (severe) postoperative complications, overall mortality, and disease-specific mortality. Postoperative complications were defined as all reported postoperative complications (anastomotic leakage, surgical site infection, etc.) after a (sub)total gastrectomy for gastric cancer according to the Clavien–Dindo grading system [18]. Severe postoperative complications were defined as complications of Clavien–Dindo grade ≥III [18].

### 2.3. Assessment of Methodological Quality

Methodological quality of all included studies was assessed using the Quality in Prognosis Studies (QUIPS) tool by two independent researchers (R.B.d.B. and A.S.B.) [19]. The QUIPS includes bias assessment on a 3-point scale in 6 domains: Study participation, study attrition, prognostic factor measurement, outcome measurement, study confounding, and statistical analysis and reporting. The applied definitions of the 3-point scale per domain are described in the supplementary material. Disagreements were resolved through discussion between the two researchers. No articles were excluded based on the results of the quality assessment.

### 2.4. Statistical Analysis

Odds ratios (OR) for postoperative complications and hazard ratios (HR) for overall mortality and disease-specific mortality with their associated variances (95% confidence intervals (95% CI) or standard errors (SE)) were obtained directly from the publication where possible. When both estimates from univariable and multivariable analyses were available, the estimates obtained by the multivariable analyses were used for further analyses. If no univariable or multivariable effect size were reported by the study, univariable ORs were calculated based on the reported numbers of events, and univariable HRs were calculated according to previously described methods [20]. Pooled estimates with 95% CIs were calculated by the random effects models for all postoperative complications, severe postoperative complications, overall mortality, and disease-specific mortality separately. Heterogeneity of the included studies was assessed by I squared values, with 0–40% considered as nonrelevant heterogeneity, 30–60% as moderate heterogeneity, 50–90% as substantial heterogeneity, and 75–100% as considerable heterogeneity [21]. Additionally, prediction intervals were calculated by random effects models to facilitate the clinical interpretation of the heterogeneity [22]. The prediction interval reflects the variation in treatment effects over different settings, including what effect is to be expected in future patients [22]. Because of the wide range of definitions of low muscle mass, we performed a sensitivity analysis including only the studies with a similar definition of low muscle mass. In this way, the effect of the wide range of definitions could be assessed. Publication bias was assessed using a visual representation of the data in a funnel plot. All statistical analyses were performed with RevMan 5.3 software (The Nordic Cochrane Centre, The Cochrane Collaboration, Copenhagen, Denmark) and R software for statistical computing version 3.1.2 (RStudio Team (2015). RStudio: Integrated Development for R. RStudio, Inc., Boston, MA, “metafor” and “metamisc” packages).

## 3. Results

### 3.1. Study Selection

**Figure 1:**
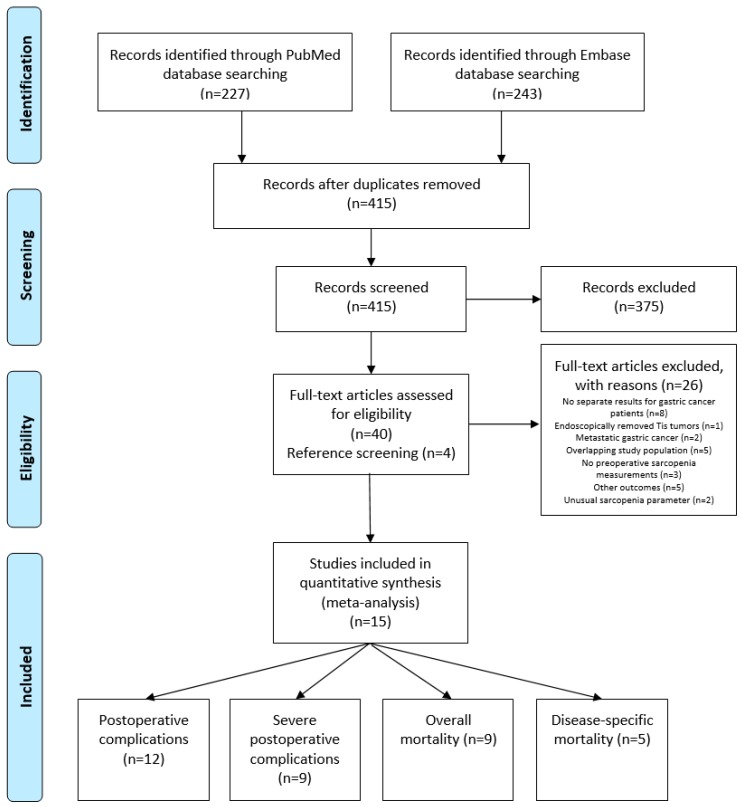
Preferred Reporting Items for Systematic Reviews and Meta-Analyses (PRISMA) flow diagram of literature search.

After removal of duplicates, a total of 415 studies reporting on low muscle mass as measured on CT scans and gastric cancer were identified. After title and abstract and full text screening, 15 studies were eligible for inclusion, reporting on 4887 patients (Figure 1). The association between low muscle mass and postoperative complications was reported in 12 studies [12,13,23,24,25,26,27,28,29,30,31,32] of which 9 studies reported data on severe postoperative complications (Clavien–Dindo grade ≥III) [12,23,24,25,26,27,28,31,32]. Overall mortality was reported as an outcome measure in 9 studies [24,25,26,28,31,33,34,35], and 5 studies reported on disease-specific mortality [28,31,33,34,35]. 

### 3.2. Study Characteristics

Most studies were retrospective cohort studies (67%) [12,13,24,25,26,27,28,31,33,34], although 5 were prospective in nature (33%) [23,29,30,32,35]. The majority of the studies included more men than women, with a percentage of male patients varying from 35% to 84%. In total, 4887 patients were included in the systematic review. The reported prevalence of low muscle mass in the included studies varied between 9% and 62%. An overview of the study characteristics is included in Table 1.

### 3.3. CT-Based Assessment of Muscle Mass

In all studies, CT-based assessment of muscle mass was performed in patients prior to surgery [12,13,23,24,25,26,27,28,29,30,31,32,33,34,35]. Thirteen studies (87%) calculated the total two-dimensional skeletal muscle area at the level of the third lumbar vertebra [12,23,24,25,27,28,29,30,31,32,33,34,35]. The areas of interest were determined either automatically, or by one or multiple observers. Several normalization techniques of the skeletal muscle area were applied, of which the normalization to the squared height of the patient was the most common (93%) [12,13,23,24,26,27,28,29,30,31,32,33,34,35]. In two studies, only the muscle area of the psoas muscle on the third lumbar level was measured, which was normalized to the squared height [13,26]. Only one study normalized the skeletal muscle area to the total body surface area [25]. Subsequently, patients were frequently categorized in low muscle mass versus normal muscle mass, as based on literature-based [12,13,23,24,27,29,30,32,33] or data-dependent cutoff values [25,26,28,31,34,35]. All cutoff values were sex-specific, and two studies used different muscle mass cutoff values for obese and nonobese patients (body mass index <25 kg/m^2^ and ≥25 kg/m^2^) [12,24].

### 3.4. Risk of Bias

**Figure 2:**
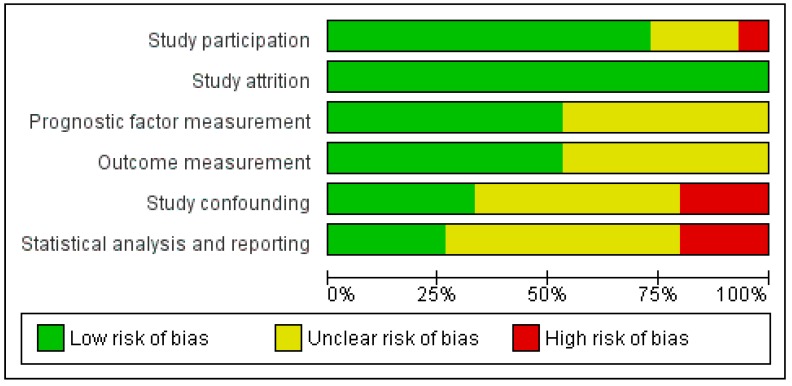
Overview of risk of bias of the included studies after Quality in Prognosis Studies (QUIPS) assessment.

Quality assessment revealed that nine (60%) studies were consecutive cohort studies with low risk of bias regarding study participation [12,23,24,26,27,29,30,32,33,34,35]. Detailed descriptions of prognostic factor measurement [12,23,24,28,30,32,33] and outcome measurement [12,23,27,29,30,31,35] were individually available in 47% of the included studies. Furthermore, only a few studies reported sufficient details on the domains of study confounding (five studies, 33%) [27,31,32,33] to be able to be qualified as having low risk of bias. Only four studies (27%) performed adequate statistical analysis and reporting to be qualified as having low risk of bias in this domain [27,31,32,33] (Figure 2). The majority of the studies (73%) reported results of multivariable analyses, of which the included covariables in the multivariable analyses are included in Table 2 [24,25,26,28,29,30,31,32,33,34,35]. The duration of follow-up varied from 1 to 64 months after surgery [23,30]. The quality of the included studies in general was thus mediocre. 

Visual inspection of the funnel plots demonstrated asymmetry towards both positive (overall mortality) and negative estimates (severe postoperative complications) of low muscle mass in relation to outcomes, indicating some level of small-study effects or publication bias (Supplementary Figures S2–S5).

### 3.5. Postoperative Complications

**Figure 3:**
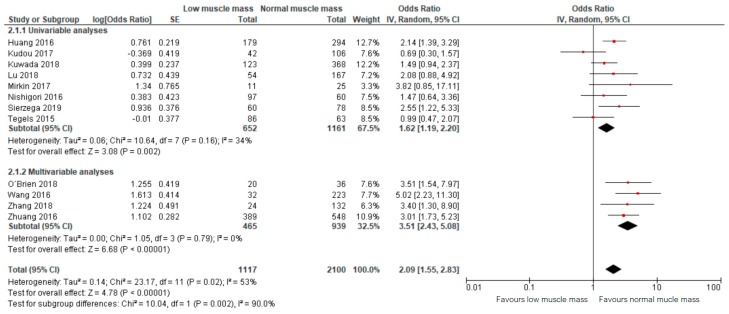
Forest plots of univariable and multivariable odds ratios for postoperative complications for gastric cancer patients with low muscle mass versus normal muscle mass.

Meta-analysis of 12 studies, including a total of 2100 patients, showed that preoperative low muscle mass (all definitions) as assessed based on CT scans was significantly associated with higher odds of all postoperative complications combined (OR: 2.09, 95% CI: 1.55–2.83, Figure 3) [12,13,23,24,25,26,27,28,29,30,31,32]. The corresponding 95% prediction interval was 0.91–4.67. In this overall analysis, substantial heterogeneity was presented, reflected by an I squared of 53% [36]. Within the results of the univariable and multivariable analyses subgroups, the heterogeneity was smaller (34% and 0%, respectively, Figure 3).

**Figure 4:**
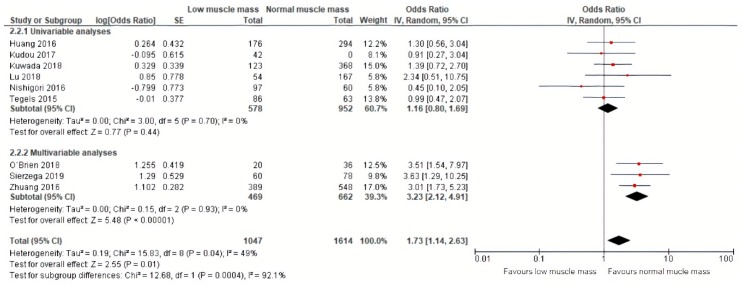
Forest plots of univariable and multivariable odds ratios for severe postoperative complications for gastric cancer patients with low muscle mass versus normal muscle mass.

Pooled analysis of data of nine studies, including 1614 patients, on severe postoperative complications (Clavien–Dindo grade ≥III) demonstrated a similar effect of preoperative low muscle mass on severe postoperative complications (OR: 1.73, 95% CI: 1.14–2.63, Figure 4) [12,23,24,25,26,27,28,31,32]. The corresponding 95% prediction interval was 0.70–4.02. Substantial heterogeneity was present in the overall analysis, as demonstrated by an I squared of 49% [36]. Within the results of the univariable and multivariable subgroups, heterogeneity was absent (0%, Figure 4). 

### 3.6. Mortality

**Figure 5:**
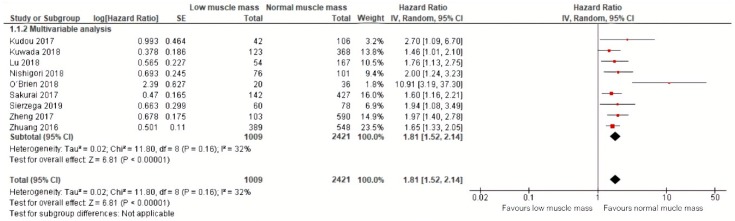
Forest plots of univariable and multivariable hazard ratios for overall mortality for gastric cancer patients with low muscle mass versus normal muscle mass.

Meta-analysis of nine studies including a total of 2421 patients with a follow-up ranging from 30 months to 64 months demonstrated that low muscle mass was significantly associated with an 81% increased mortality (HR: 1.81, 95% CI: 1.52–2.14) [24,25,26,28,31,32,33,34,35]. The corresponding 95% prediction interval was 1.52–1.94. Additional calculations showed an I squared test of 32% [36] (Figure 5). All effect sizes included in this meta-analysis were obtained by multivariable analyses.

**Figure 6:**
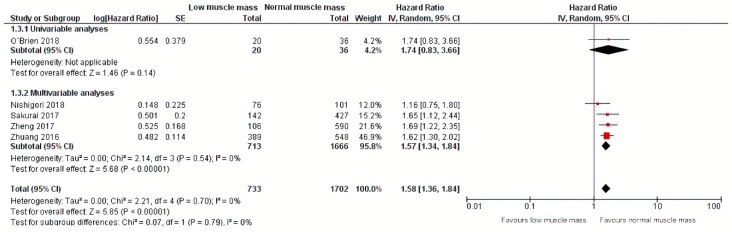
Forest plots of univariable and multivariable hazard ratios for disease-specific mortality for gastric cancer patients with low muscle mass versus normal muscle mass.

Regarding disease-specific mortality, a meta-analysis of one univariable analysis and four multivariable analyses including 1702 patients showed that low muscle mass was also significantly related to an increase of 53% in disease-specific mortality (HR: 1.58, 95% CI: 1.36–1.84) [27,28,31,34,35] with a corresponding 95% prediction interval of 1.36–1.84. Additional calculations showed an I squared test of 0% [36] (Figure 6). Subgroup analyses for studies that performed univariable and multivariable analyses are also depicted in Figure 6.

### 3.7. Sensitivity Analysis

To assess the effect of the different definitions of CT-assessed muscle mass we performed a sensitivity analysis including only the studies with a similar definition of muscle mass (Hounsfield Units from −29 or −30 to +150). Hence, we excluded two studies that used Hounsfield Units of muscle mass from −30 to +110 [12,26] and two studies without description [13,24]. After exclusion of the four studies, low muscle mass had a stronger effect on total postoperative complications (OR: 2.43, 95% CI: 1.83–3.24, Supplementary Figure S6) [23,25,27,28,29,30,31,32]. This also accounted for the effect of low muscle mass on severe postoperative complications (OR: 2.01, 95% CI: 1.22–3.31, Supplementary Figure S7) [23,25,27,28,31,32]. A minimal effect was shown in the overall mortality analysis after exclusion of the four studies (OR: 1.80, 95% CI: 1.47–2.21, Supplementary Figure S8) [25,28,31,32,33,34,35]. The meta-analysis of disease-specific mortality did not include any study with a different muscle mass definition.

## 4. Discussion

This systematic review and meta-analysis demonstrated that low muscle mass assessed using CT scans has a significant predictive effect on postoperative complications and mortality in gastric cancer patients. Gastric cancer patients with low muscle mass have higher odds of (severe) postoperative complications and a higher overall and disease-specific mortality. These findings can aid individual preoperative risk assessment, which might help surgeons to select the right patients for surgery and provide more personalized counseling of surgical benefit and risks for patients. Furthermore, the results might provide rationale for the use of preoperative training, nutritional programs, or dedicated pharmacotherapy in gastric cancer patients to enhance a patient’s physical condition and ultimately to improve postoperative outcomes. 

Our findings correspond with previous systematic reviews in patients with other malignancies. CT-based assessment of low muscle mass was associated with an increase of postoperative complications in colorectal cancer patients and an increased overall mortality in gastrointestinal and hepatopancreatobiliary cancer patients [37]. In esophageal cancer patients, a systematic review and meta-analysis showed that low muscle mass, assessed with CT, dual-energy-X-ray absorptiometry (DXA), or bioelectrical impedance analysis (BIA), resulted in a higher incidence of pulmonic complications and increased overall mortality [38].

The current systematic review and meta-analysis only focused on CT-based assessment of low muscle mass. However, other noninvasive techniques for body composition assessment are also available; for example, BIA and DXA. DXA is a common instrument to measure muscle mass using radiation. However, different DXA brands have shown inconsistent results, and measurements can be influenced by hydration status [11]. BIA is a practical tool for body composition assessment, which is based on a small electrical current traveling through the body. However, studies reporting on accuracy of BIA have been inconsistent, limiting its application in clinical practice [39]. Because CT scans are part of the routine (re)staging process in gastric cancer patients, it is a suitable tool for muscle mass quantification without subjecting patients to additional ionizing radiation. 

Sarcopenia in cancer patients might be caused by several factors; for example, inactivity, malnutrition, and cancer-related cytokines [40]. Another factor that might influence sarcopenia in gastric cancer patients is tumor (lymph) nodes metastasis (TNM) stage. As more advanced tumors with a higher TNM stage are likely to cause more obstruction, patients with high TNM stages might be more prone to being sarcopenic due to limited oral intake and absorption. In addition, more advanced tumors may also produce more cancer-related cytokines that induce sarcopenia. This might influence the observed association between low muscle mass and postoperative outcomes in this study. In the analyses of (severe) postoperative complications, univariable analyses were prevalent, and confounding by TNM stage might therefore influence the obtained results of the meta-analyses. However, for the analyses on overall mortality, nearly all studies reported multivariable results and all studies included tumor stage as a covariable in their multivariable analyses. So the confounding effect of TNM stage is limited in the mortality analyses. Furthermore, high tumor stage is correlated with poor survival, regardless of low muscle mass [8]. To limit this bias, we created a more homogenous tumor stage group by excluding in situ tumors and palliative gastric cancer.

Considerable variety exists in the current definition of sarcopenia, even when only including CT-based assessment of low muscle mass [41]. After exclusion of studies that used different Hounsfield units for muscle mass, the effect of low muscle mass on (severe) postoperative complications increased in the sensitivity analysis. Most studies based their assessment of muscle mass on the two-dimensional skeletal muscle area at the level of the third lumbar vertebra, which is normalized to the squared height of the patient. However, some studies only used the area of the psoas muscle instead of the full musculature. The full cross-sectional muscle area at the third lumbar vertebra is known to be a better estimate of the total body musculature than the psoas muscle area alone [42]. This indicates that there is a need for a universal definition of low muscle mass for CT-based measurements, as heterogeneity in the applied definitions hampers comparability of the results obtained by published studies. 

One study compared several cutoff values for low muscle mass. They concluded that the definition by Martin et al. would be the preferred cutoff point to predict overall survival: A skeletal muscle index (SMI) of 53.0 cm^2^/m^2^ for male patients with a BMI ≥25 kg/m^2^, SMI of 43.0 cm^2^/m^2^ for male patients with a BMI <25 kg/m^2^, and SMI of 41.0 cm^2^/m^2^ for female patients [14,33]. 

In 2018, the definition and diagnosis of sarcopenia was revised by the European Working Group on Sarcopenia in Older People (EWGSOP). It was recommended to use an algorithm to diagnose sarcopenia and to perform a severity assessment. The SARC-F (strength, assistance in walking, rise from a chair, climb stairs, falls) questionnaire aims at finding sarcopenic patients [42]. Subsequently, the committee advised use of hand grip strength to identify low muscle strength. To generate evidence for low-quantity and -quality muscle in research and high-risk patients, they suggested DXA, MRI, or CT scans. Physical performance tests are advised by the committee to assess sarcopenic severity [11].

Most studies included in the current meta-analysis were conducted in Asia. As such, the generalizability of the results to the Western population might be impaired. Moreover, different durations of follow-up have been reported in the included articles. This might have influenced the conclusions obtained in the analyses of overall and disease-specific mortality. Lastly, the quality of most included studies was mediocre based on the QUIPS criteria. As such, improvements should be made in the methodology and reporting of studies on CT-based muscle mass assessment.

In order to facilitate the introduction of CT assessment of muscle mass to clinical practice, an accurate automated contouring tool would be desirable, which would obviate the pragmatic need to limit measurement to the L3 level, and instead compute actual muscle volume over the whole three-dimensional volume of the patient’s body. Moreover, a universal definition of (CT-based assessment of) low muscle mass should be used in future studies to be able to more effectively compare results. According to the most recent consensus on sarcopenia, physical performance tests may be included for more accurate assessment of sarcopenia. Furthermore, a recent study demonstrated that low muscle mass is also a risk factor for developing dose-limiting toxicity during neoadjuvant therapy in gastric cancer patients [43]. Lastly, the change of body composition parameters over time (for example, during neoadjuvant therapy or postoperative changes) might also be an interesting tool for outcome prediction. 

## 5. Conclusions

In conclusion, this systematic review and meta-analysis demonstrated that CT-based assessment of low muscle mass, even without functional assessment, is associated with increased odds of (severe) postoperative complications in gastric cancer patients. Moreover, gastric cancer patients with low muscle mass have an increased overall and disease-specific mortality compared to patients with normal muscle mass. However, the included studies reported variable assessment methods and cutoff values for low muscle mass. A universal definition of low muscle mass as measured on CT scans is preferable for further research. 

## Figures and Tables

**Table 1:** Details of included studies.

Author, Year, Continent	Study Design	No. of Included Patients	Mean Age (Years)	Sex (% Male)	Tumor Stage	Type of Gastrectomy (Total Versus Partial and Surgical Approach)	Mean Follow-up Time (Months)	Time of Muscle Mass Assessment	Level of Assessment of Muscle Mass	Definition of Muscle Mass on CT in HU	Definition of Low Muscle Mass (cm^2^/m^2^)	Normalization	No. of Patients with Low Muscle Mass (%)
Tegels, 2015, Europe	RCS	152	70	59%	0—3% Ia—8% Ib—16% II—15% IIIa—15% IIIb—11% IV—32% Not reported—1%	Total: 43 Subtotal: 104 Gastroenterostomy: 17 Laparotomy: 16 Approach: NR	6	Preoperative	L3 (caudal level)	−30 to +110	BMI < 25.0 kg/m2: Male < 43, Female < 41 BMI ≥ 25.0 kg/m2: Male < 53, Female < 41	SMA/height squared	86 (58%)
Huang, 2016, Asia	PCS	470	65	77%	I—35% II—22% III—43%	Total: 164 Subtotal: 306 Laparoscopic: 198 Open: 742	1	Preoperative	L3 (caudal level)	−29 to +150	Male < 40.8, Female < 34.9	SMA/height squared	49 (10%)
Nishigori, 2016, Asia	RCS	157	66	66%	I—59% II—17% III—17% IV—1%	Total laparoscopic: 157	NR	Preoperative	L3	−29 to +150	Male < 52.4, Female < 38.5	SMA/height squared	97 (62%)
Wang, 2016, Asia	PCS	255	65	84%	I—32% II—19% III—49%	Total: 85 Subtotal: 170 Open: 209 Laparoscopic: 46	1	Preoperative	L3 (caudal level)	−29 to +150	Male < 36.0, Female < 29.0	SMA/height squared	23 (9%)
Zhuang, 2016, Asia	RCS	937	64	78%	I—29% II—23% III—48%	Total: 337 Subtotal: 600 Approach: NR	62	Preoperative	L3 (caudal level)	−29 to +150	Male < 40.8, Female < 34.9	SMA/height squared	389 (42%)
Kudou, 2017, Asia	RCS	148	65	72%	NR	Total: 143 Proximal: 5 Thoracotomy or thoracoscopy: 9	60	Preoperative	L3	NR	BMI < 25.0 kg/m2: Male < 43, Female < 41; BMI ≥ 25.0 kg/m2: Male < 53, Female < 41	SMA/height squared	42 (28%)
Sakurai, 2017, Asia	RCS	569	67	70%	I—46% II—21% III—22% IV—58%	Total: 203 Partial: 366 Open: 378 Laparoscopy: 191	32	Preoperative	L3	−29 to +150	Male < 43.2, Female < 34.6	SMA/height squared	142 (25%)
Mirkin, 2017, North America	RCS	36	64	35%	NR	Total: 26 Subtotal: 10 Robot: 36	18	Preoperative: Before and after neoadjuvant therapy	L3 (caudal level)	NR	Male < 5.45, Female < 3.85	Psoas muscle area/height squared	12 (33%)
Zheng, 2017, Asia	PCS	924	61	76%	T4—57.1% N+—47.6%	Radical gastrectomy: 924 Approach: NR	36	Preoperative	L3 (vertebral spines visible)	−29 to +150	Male < 32.5, Female < 28.6	SMA/height squared	103 (11%)
Kuwada, 2018, Asia	RCS	491	68	71%	≥III—22%	Total: 26 Distal:192 Partial: 38 Other: 44 Approach: NR	NR	Preoperative	L3	−30 to +150	Male < 69.7, Female < 54.2	SMA/BSA	123 (25%)
Lu, 2018, Asia	RCS	221	62	77%	I—31% II—24% III—44%	Total: 111 Subtotal: 110 Open: 37 Laparoscopy: 184	64	Preoperative	L3 (vertebral spines visible)	−30 to +110	Male < 5127, Female < 3443	Psoas muscle area/height squared	NR
Nishigori, 2018, Asia	RCS	177	<65: 33%	72%	II—56% III—44%	Total: 69 Distal: 106 Proximal: 2 Open: 59 Laparoscopy: 116 Robot: 2	58	Preoperative	L3	−29 to +150	Male < 36.0–53.0, Female < 29.0–41.0	SMA/height squared	76 (43%)
O’Brien, 2018, Europe	RCS	56	69	73%	0—13% Ia—20% Ib—13% IIa—11% IIIa—1% IIIb—16% IIIc—11%	Total: 34 Distal: 12 Proximal: 10 Approach: NR	40	Preoperative	L3	−30 to +150	Male < 52.4, Female < 38.5	SMA/height squared	20 (36%)
Zhang, 2018, Asia	PCS	156	59	74%	I—31% II—17% III—52%	Total: 45 Subtotal: 111 Open: 156	NR	Preoperative	L3 (transverse process visible)	−29 to +150	Male < 40.8, Female < 34.9	SMA/height squared	24 (15%)
Sierzega, 2019, Europe	PCS	138	63	58%	I—14% II—22% III—46% IV—18%	Total: 77 Subtotal distal: 61 Laparotomy: 138	30	Preoperative	L3	−29 to +150	Male < 52.4, Female < 38.5	SMA/height squared	60 (43%)

Note: BSA, body surface area; HU, Hounsfield Unit; L3, third lumbar vertebra; NA, not available based on the results reported by the study; NN, nonsarcopenic nonobesity; NO, nonsarcopenic obesity; NR, not reported by the study; PCS, prospective cohort study; RCS, retrospective cohort study; SMA, skeletal muscle area; SN, sarcopenic nonobesity; SO, sarcopenic obesity; TNM stage, tumor (lymph) nodes metastasis stage.

**Table 2:** Overview of covariables included in the multivariable analyses of the included studies per outcome (postoperative complications, severe postoperative complications, overall mortality, disease-specific mortality).

Author and Year	Included Covariables in Analyses			
	Postoperative Complications	Severe Postoperative Complications	Overall Mortality	Disease-Specific Mortality
Wang, 2016	Diabetes			
Zhuang, 2016		Diabetes	Age, sex, TNM stage, type of resection, severe complications, neoadjuvant therapy	Histology, TNM stage, type of resection, operative time, adjuvant therapy, age, sex, BMI, hypoproteinemia, anemia, tumor size, tumor location, lymphovascular invasion, combined resection
Kudou, 2017			Age, tumor location, T stage, N stage, blood loss	
Sakurai, 2017			Age, histology, T stage, N stage, type of gastrectomy, intra-abdominal infection	Histology, T stage, N stage, type of gastrectomy
Zheng, 2017			Age, BMI, T stage, N stage, albumin, ASA score, adjuvant chemotherapy	BMI, T stage, N stage, sarcopenia, albumin, ASA score, adjuvant therapy
Kuwada, 2018			Age, comorbidity, histology, T score, N score, operation procedure, operation time, blood loss, postoperative complications	
Lu, 2018			Total psoas gauge, Hounsfield unit average calculation, tumor stage	
Nishigori, 2018			Sex, age, serum albumin, creatinine clearance, BMI, Charlson comorbidity index, and clinical stage	Sex, age, serum albumin, creatinine clearance, BMI, Charlson comorbidity index, and clinical stage
O’Brien, 2018		Sex	Body mass index, tumor stage	
Zhang, 2018	Retinol-binding protein, myosteatosis			
Sierzega, 2019		Age, BMI, NRS2002, respiratory comorbidity, diabetes, ASA score, type of gastrectomy, lymph node dissection, combined organ dissection, curative resection, perioperative chemotherapy	ASA score, TNM stage, curative resection, type of gastrectomy, major complications	

## Supplementary Materials

The following are available online at https://www.mdpi.com/2077-0383/9/1/199/s1, Table S1: Overview of search strategy, Table S2: Applied definitions of the 3-point scale per domain of QUIPS, Figure S1: Overview of the risk of bias score of the included studies following QUIPS, Figure S2: Funnel plot of included studies reporting on postoperative complications, Figure S3: Funnel plot of included studies reporting on severe postoperative complications, Figure S4: Funnel plot of included studies reporting on overall mortality, Figure S5: Funnel plot of included studies reporting on disease-specific mortality.
