# Supplementary material for: The Predictive Value of Low Muscle Mass as Measured on CT Scans for Postoperative Complications and Mortality in Gastric Cancer Patients: A Systematic Review and Meta-Analysis
Source: J Clin Med. 2020 Jan 11;9(1):199. doi: 10.3390/jcm9010199 (PMC7019480; doi:10.3390/jcm9010199)
Supplement: Supplementary file 1 [file jcm-09-00199-s001.pdf]

Journal of Clinical Medicine

MDPI

Review

The Predictive Value of Low Muscle Mass as Measured on CT Scans for Postoperative Complications and Mortality in Gastric Cancer Patients: A Systematic Review and Meta-Analysis

Alicia S. Borggreve [1,2,+], Robin B. den Boer [1,+], Gijs I. van Boxel [1], Pim A. de Jong [3], Wouter B. Veldhuis [3], Elles Steenhagen [4], Richard van Hillegersberg [1] and Jelle P. Ruurda [1,*]

Supplementary Materials:

Table S1. Overview of search strategy.

#1	#2	#3	#4
Stomach	Cancer*	"Muscle mass"	"Computed Tomography"
Gastric	Neoplas*	"Skeletal muscle"	CT
Cardia	Malignan*	"Psoas muscle"	CAT
	Tumor*	Myopenia	Scan*
	Tumour*	Sarcopen*	
	Oncolog*	"Total muscle area"	
	Carcinoma*	"Body composition"	
	"Squamous cell carcinoma*"	"Total Psoas Area"	
	Adenocarcinoma*	"Muscle wasting"	
		"Muscle attenuation"	
		"Muscular atrophy"	
**MeSH-terms:**<br>- Stomach<br>-"Stomach neoplasms"<br>- Cardia<br>-"Gastrointestinal neoplasms"	**MeSH-terms:**<br>- Neoplasms<br>- Carcinoma<br>-"Neoplasms squamous cell"<br>-Adenocarcinoma	**MeSH-terms:**<br>- Sarcopenia<br>-"Muscular atrophy"<br>-"Psoas muscle"<br>-"Muscle, skeletal"<br>-"Body composition"	**MeSH-terms:**<br>-"Tomography, X-Ray Computed"<br>- Radiography
**Emtree-terms:**<br>- Stomach (exp)	**Emtree-terms:**<br>- 'Malignant neoplasm' (exp)	**Emtree-terms:**<br>- 'Muscular atrophy'<br>- 'Skeletal muscle' (exp)<br>- 'Body composition' (exp)	**Emtree-terms:**<br>- 'Computer assisted tomography'<br>- 'Digital imaging' (exp)

Table S2. Applied definitions of the 3-point scale per domain of QUIPS.

	Low Risk of Bias	Moderate Risk of Bias	High Risk of Bias
Study participation	Adequate reporting of patient cohort. In- and exclusion criteria mentioned clearly. Baseline study sample is well described for important patient characteristics.	Moderate reporting of patient cohort and in- and exclusion criteria. Moderate description of baseline study sample for important patient characteristics.	Inferior reporting of patient cohort and in- and exclusion criteria. Inferior description of baseline study sample for important patient characteristics.
Study attrition	Adequate reporting of response rate, reasons for loss to follow up, and attempts to collect data from patients who dropped out of study.	Moderate reporting of response rate, reasons for loss to follow up, and attempts to collect data from patients who dropped out of study.	Inferior reporting of response rate, reasons for loss to follow up, and attempts to collect data from patients who dropped out of study.
Prognostic factor measurement	Adequate reporting of the method of muscle mass assessment. Blinded and experienced investigator. Cutoff value for low muscle mass was based on large patient cohorts and not data-dependent.	Moderate reporting of the method of muscle mass assessment. Blinded and experienced investigator. A data-dependent cutoff value was used for the definition of low muscle mass.	Inferior reporting of the method of muscle mass assessment. Not-blinded or inexperienced investigator. A data-dependent cutoff value was used for the definition of low muscle mass.
Outcome measurement	Adequate reporting of definition of outcomes, duration of follow-up, and method of outcome measurement.	Moderate reporting of definition of outcomes, duration of follow-up, and method of outcome measurement.	Inferior reporting of definition of outcomes, duration of follow-up, and method of outcome measurement.
Study confounding	Adequate accounting for possible confounders: BMI, gender, smoking/alcohol, TNM stage, performance score, histological type, therapy (surgery, chemotherapy), comorbidity, tumor location, regression grade in case of chemotherapy, nutritional status.	Moderate accounting for possible confounders: BMI, gender, smoking/alcohol, TNM stage, performance score, histological type, therapy (surgery, chemotherapy), comorbidity, tumor location, regression grade in case of chemotherapy, nutritional status.	Inferior accounting for possible confounders: BMI, gender, smoking/alcohol, TNM stage, performance score, histological type, therapy (surgery, chemotherapy), comorbidity, tumor location, regression grade in case of chemotherapy, nutritional status.
Statistical analysis and reporting	Meta-analysis with univariable analysis only.	Meta-analysis with multivariable analysis with covariables included that were significant in the univariable analysis ($p < 0.05$).	Meta-analysis with multivariable analysis with covariables included that were significant in the univariable analysis ($p < 0.1$).

	Study participation	Study attrition	Prognostic factor measurement	Outcome measurement	Study confounding	Statistical Analysis and reporting
Tegels 2015	Low	Low	Low	Low	High	High
Huang 2016	Low	Low	Low	Low	Moderate	Moderate
Nishigori 2016	Low	Low	Moderate	Low	Low	Low
Wang 2016	Low	Low	Low	Low	Moderate	Moderate
Zhuang 2016	Moderate	Low	Moderate	Low	Low	Low
Kudou 2017	Low	Low	Low	Moderate	High	High
Sakurai 2017	Low	Low	Moderate	Moderate	Moderate	Moderate
Mirkin 2017	Moderate	Low	Moderate	Moderate	High	High
Zheng 2017	Low	Low	Moderate	Low	Moderate	Moderate
Kuwada 2018	Moderate	Low	Moderate	Moderate	Moderate	Moderate
Lu 2018	Low	Low	Moderate	Moderate	Moderate	Moderate
Nishigori 2018	Low	Low	Low	Moderate	Low	Low
O´Brien 2018	High	Low	Low	Moderate	Moderate	Moderate
Zhang 2018	Low	Low	Moderate	Low	Moderate	Moderate
Siezerga 2019	Low	Low	Low	Moderate	Low	Low

Figure S1. Overview of the risk of bias score of the included studies following QUIPS.

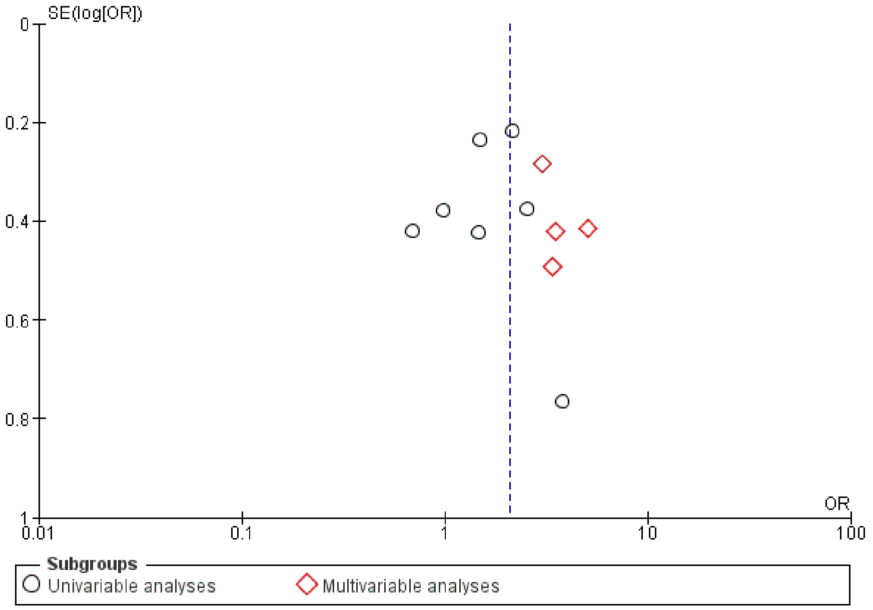

Figure S2. Funnel plot of included studies reporting on postoperative complications.

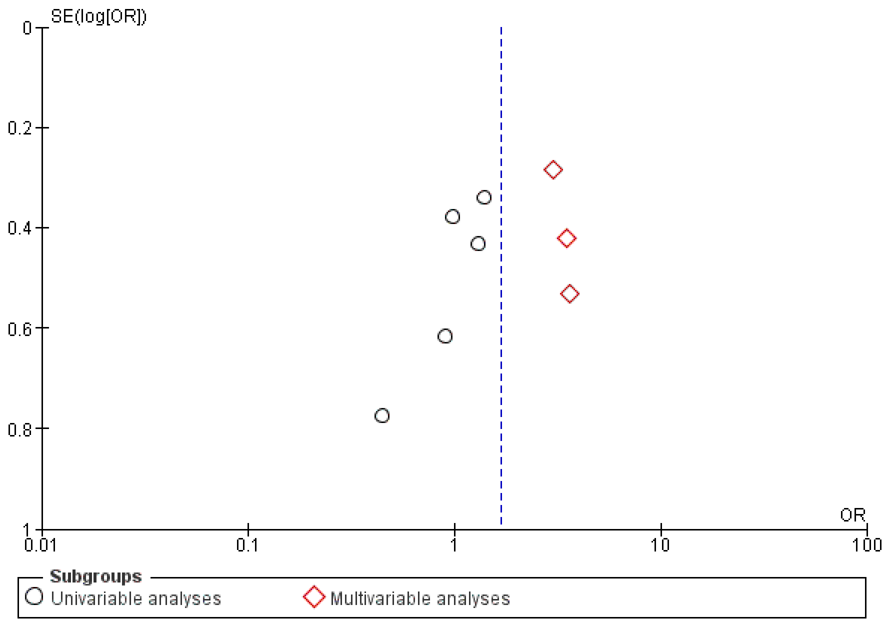

Figure S3. Funnel plot of included studies reporting on severe postoperative complications.

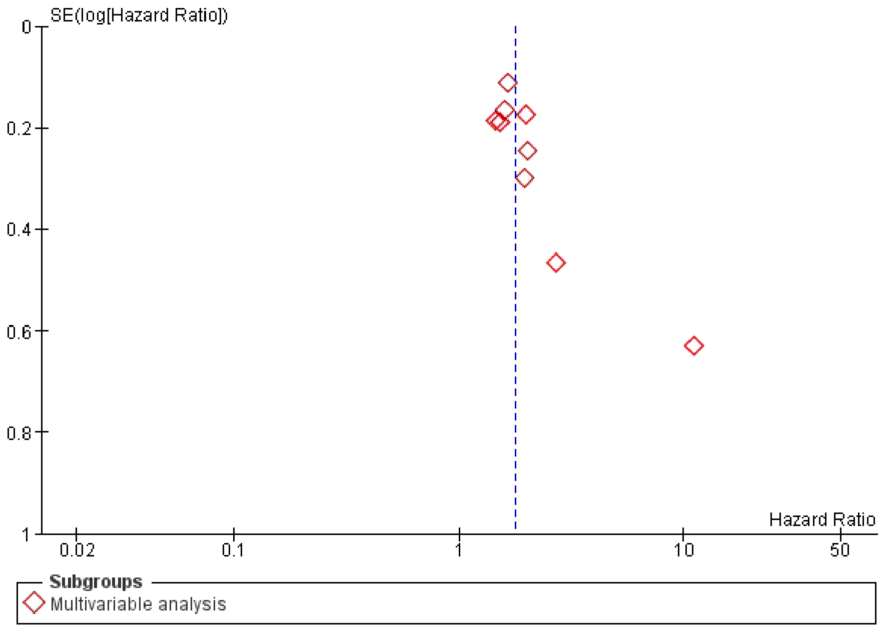

Figure S4. Funnel plot of included studies reporting on overall mortality.

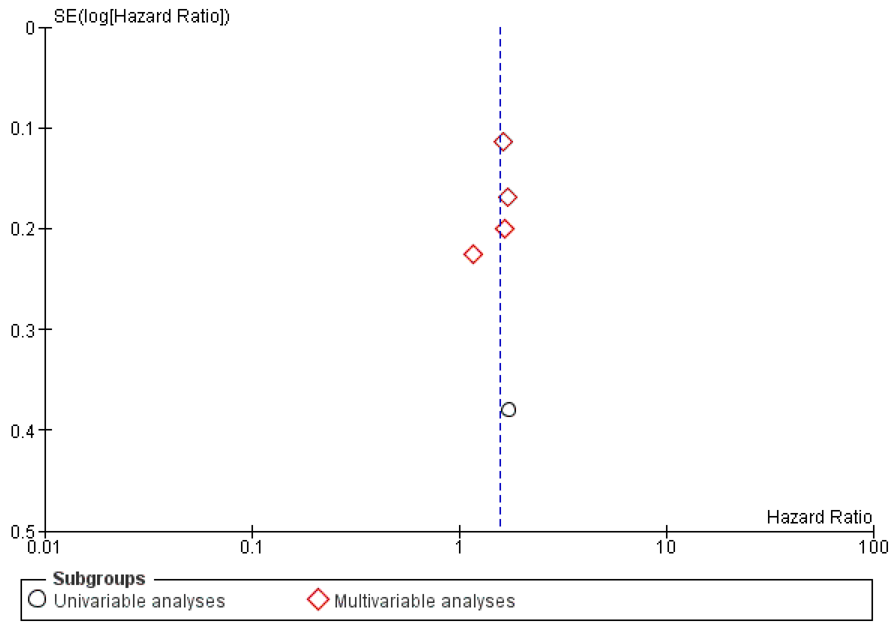

Figure S5. Funnel plot of included studies reporting on disease-specific mortality.

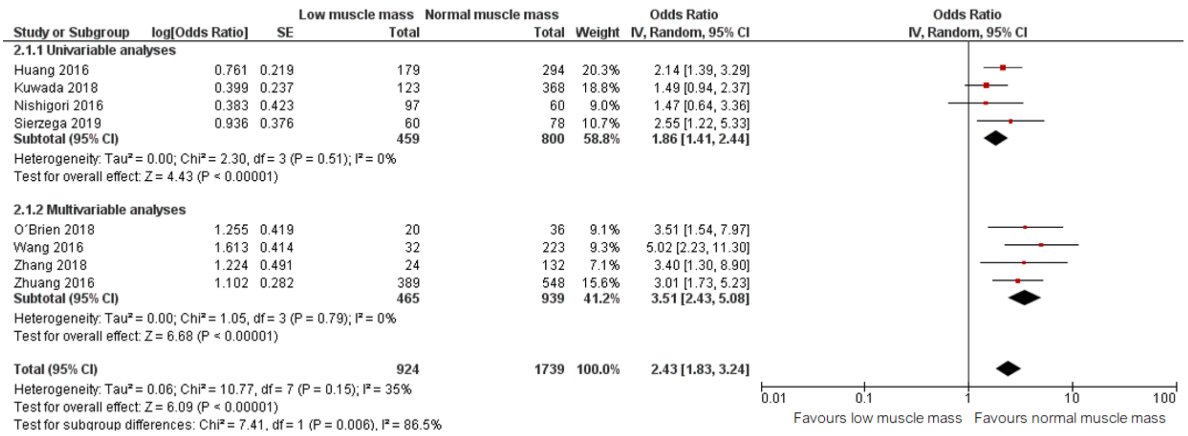

Figure S6. Sensitivity analysis: forest plots of univariable and multivariable odds ratios for postoperative complications for gastric cancer patients with low muscle mass versus normal muscle mass.

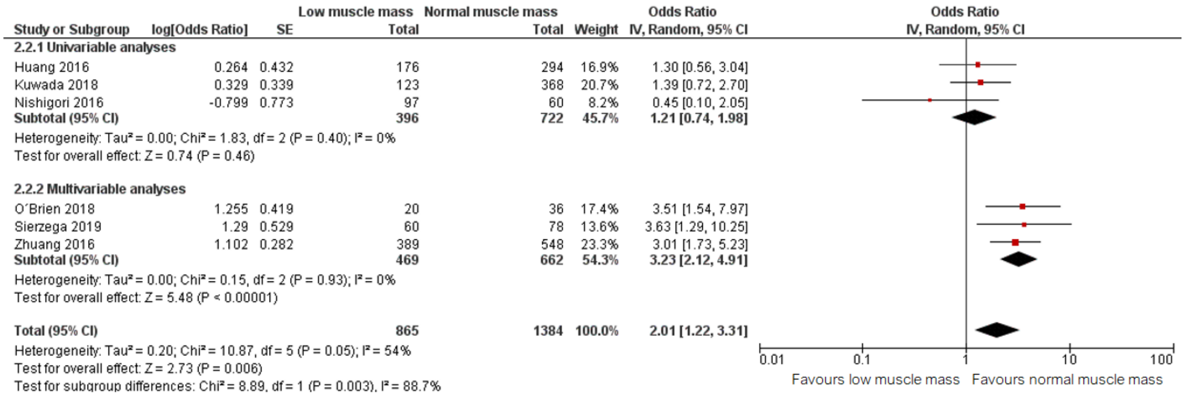

Figure S7. Sensitivity analysis: forest plots of univariable and multivariable odds ratios for severe postoperative complications for gastric cancer patients with low muscle mass versus normal muscle mass.

Study or Subgroup	log[Hazard Ratio]	SE	Low muscle mass Total	Normal muscle mass Total	Weight	Hazard Ratio IV, Random, 95% CI
**1.1.2 Multivariable analysis**						
Kuwada 2018	0.378	0.186	123	368	16.3%	1.46 [1.01, 2.10]
Nishigori 2018	0.693	0.245	76	101	11.7%	2.00 [1.24, 3.23]
O´Brien 2018	2.39	0.627	20	36	2.5%	10.91 [3.19, 37.30]
Sakurai 2017	0.47	0.165	142	427	18.4%	1.60 [1.16, 2.21]
Sierzega 2019	0.663	0.299	60	78	8.8%	1.94 [1.08, 3.49]
Zheng 2017	0.678	0.175	103	590	17.4%	1.97 [1.40, 2.78]
Zhuang 2016	0.501	0.11	389	548	24.9%	1.65 [1.33, 2.05]
**Subtotal (95% CI)**			**913**	**2148**	**100.0%**	**1.80 [1.47, 2.21]**
Heterogeneity: Tau² = 0.03; Chi² = 10.92, df = 6 (P = 0.09); I² = 45%						
Test for overall effect: Z = 5.73 (P < 0.00001)						
**Total (95% CI)**			**913**	**2148**	**100.0%**	**1.80 [1.47, 2.21]**
Heterogeneity: Tau² = 0.03; Chi² = 10.92, df = 6 (P = 0.09); I² = 45%						
Test for overall effect: Z = 5.73 (P < 0.00001)						
Test for subgroup differences: Not applicable						

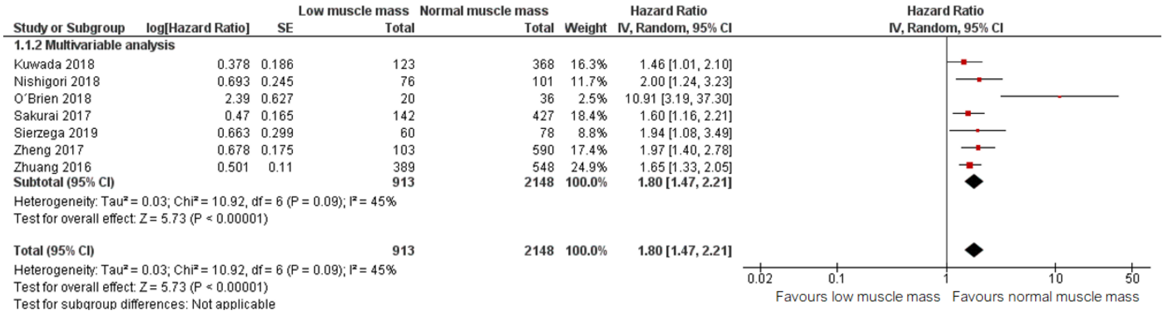

Figure S8. Sensitivity analysis: forest plots of univariable and multivariable odds ratios for overall survival for gastric cancer patients with low muscle mass versus normal muscle mass.
